# Cytoprotective effect of chlorogenic acid against hydrogen peroxide-induced oxidative stress in MC3T3-E1 cells through PI3K/Akt-mediated Nrf2/HO-1 signaling pathway

**DOI:** 10.18632/oncotarget.14747

**Published:** 2017-01-19

**Authors:** Dandan Han, Wei Chen, Xiaolong Gu, Ruixue Shan, Jiaqi Zou, Gang Liu, Muhammad Shahid, Jian Gao, Bo Han

**Affiliations:** ^1^ College of Veterinary Medicine, China Agricultural University, Beijing 100193, P. R. China

**Keywords:** chlorogenic acid, MC3T3-E1 cells, oxidative stress, Nrf2/HO-1 pathway, cytoprotection

## Abstract

Osteoporosis is a disorder of bone and its development is closely associated with oxidative stress and reactive oxygen species (ROS). Chlorogenic acid (CGA) has potential antioxidant effects and its pharmacological action in osteoblasts is not clearly understood. The present study aimed to clarify the protective effects and mechanisms of CGA on hydrogen peroxide (H_2_O_2_)-induced oxidative stress in osteoblast cells. MC3T3-E1 cells were treated with H_2_O_2_ to induce oxidative stress model *in vitro*. Cells were treated with CGA prior to H_2_O_2_ exposure, the intracellular ROS production, malondialdehyde content, nitric oxide release and glutathione level were measured. We also investigated the protein levels of heme oxygenase-1 (HO-1), the nuclear translocation of transcription factor NF-erythroid 2-related factor (Nrf2) and the phosphorylation levels of Akt in CGA-treated cells. The results showed that pretreatment of CGA could reverse the inhibition of cell viability and suppress the induced apoptosis and caspase-3 activity. Additionally, it significantly reduced H_2_O_2_-induced oxidative damage in a dose-dependent manner. Furthermore, it induced the protein expression of HO-1 together with its upstream mediator Nrf2, and activated the phosphorylation of Akt in MC3T3-E1 cells. LY294002, a PI3K/Akt inhibitor, significantly suppressed the CGA-induced Nrf2 nuclear translocation and HO-1 expression. Reduction of cell death mediated by CGA in presence of H_2_O_2_ was significantly inhibited by Zinc protoporphyrin IX (a HO-1 inhibitor) and LY294002. These data demonstrated that CGA protected MC3T3-E1 cells against oxidative damage via PI3K/Akt-mediated activation of Nrf2/HO-1 pathway, which may be an effective drug in treatment of osteoporosis.

## INTRODUCTION

Oxidative stress is a crucial initiating factor in a variety of pathological conditions including osteoporosis, resulting from excessive generation of ROS or damage of the anti-oxidative stress defense system in impairment of osteoblastic bone formation [1]. Most of studies reported that the pathogenesis of osteoporosis is associated with oxidative stress in osteoblasts [2, 3]. Overproduction of ROS disrupts the balance between oxidation and antioxidant defense systems, leading to bone loss by promoting lipid peroxidation and lowering antioxidant enzymes, inducing apoptosis of osteoblasts and inhibiting bone formation [4–6]. In terms of the possible importance of ROS in bone metabolism, it is essential for cells to effectively up-regulate antioxidants, decrease ROS generation and scavenge free radical, which may contribute to bone loss and antagonize osteoporosis.

Nuclear factor-erythroid 2-related factor 2 (Nrf2), also known as NF-E2-like 2 (NFE2L2), plays an essential role in the cellular defense against various inflammatory and oxidative stress-induced diseases, including osteoporosis, which is a transcription factor that binds to antioxidant response element and regulates the production of multiple anti-oxidative enzymes [7–11]. Under stress conditions, Nrf2 dissociates from Kelch-like ECH-associated protein-1 (Keap1) and translocates into the nucleus to activate the expression of antioxidant-responsive genes and induction of phase II detoxifying enzymes, which is considered as a major regulator of oxidant resistance and involved in the maintenance of the cellular redox homeostasis, and elimination of reactive oxidants and electrophilic agents [8, 10, 11]. Heme oxygenase-1 (HO-1), a well-known intracellular inducible phase II enzyme, is involved in defense against various oxidative-inducing agents, including LPS [9], hydrogen peroxide [12], hypoxia [13] and subsequent lethality [14]. Upon oxidative stress, HO-1 is up-regulated and enhancement of HO-1 expression is potentially important in iron homeostasis, antioxidant defense, and anti-resorption of bone [15–17]. The HO-1 gene is primarily regulated at the transcriptional level, and transcriptional regulation of HO-1 protein expression is mediated by Nrf2 activation [18]. In particular, a non-cytotoxic pharmacological agent may maximize the intrinsic anti-oxidant potential of cells and represent a promising therapeutic intervention in treating a variety of disorders related to oxidative stress and inflammation via increasing HO-1 expression [18, 19]. Indeed, PI3K/Akt is a key survival signaling pathway in governing the cellular defense system against oxidative injury, and regulating cell proliferation survival and apoptosis associated with Nrf2 activation and HO-1 induction [20, 21]. Therefore, the PI3K/Akt pathway plays a vital role in the Nrf2-mediated antioxidant response, making it a potential target of drug therapy by modulating Nrf2 as an upstream signaling molecule.

So far, numerous polyphenols have been identified and proposed as the therapeutic agents to counteract oxidative stress-related diseases, which is involved in the balance between the cellular production and elimination of free radicals [22]. CGA, a type of polyphenolic compound formed by esterification of caffeic and quinic acids, is the major active ingredient found in many traditional Chinese medicines such as *Flos Lonicerae Japonicae* and *Eucommia Ulmoides*, and it is also abundant in some fruits, dietary vegetables, and daily beverages like coffee, pineapple, beans, strawberries and apples [23]. Previous reports have demonstrated that CGA exhibits a wide range of diverse pharmacological effects, including anti-inflammatory, anti-oxidative, and anti-carcinogenic activities [3, 24, 25]. However, the antioxidant effects and molecular mechanisms of CGA in osteoblasts have not been elucidated yet. Therefore, the present study established an *in vitro* experimental model of osteoblasts exposed to H_2_O_2_ to evaluate the effect of CGA on H_2_O_2_-induced oxidative stress of the osteoblast MC3T3-E1 cells, and investigate whether CGA conferred an antioxidant defense capacity via modulation of PI3K/Akt activity and activation of Nrf2 and HO-1 expressions.

## RESULTS

### H_2_O_2_ inhibits MC3T3-E1 cells proliferation and induces apoptosis

**Figure 1:**
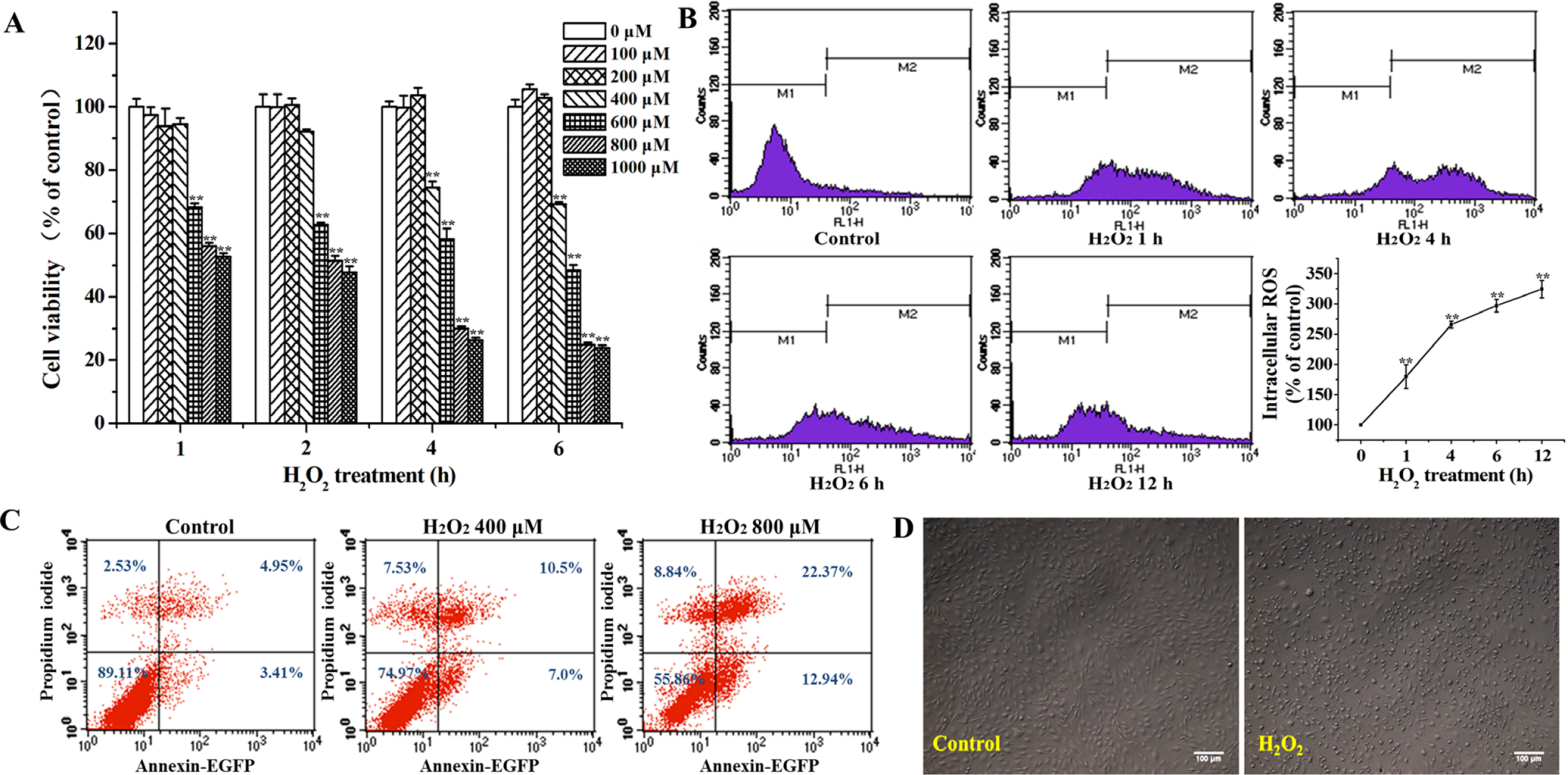
Effects of H_2_O_2_ on the apoptosis of MC3T3-E1 cells (**A**) MC3T3-E1 cells were treated with various concentrations of H_2_O_2_ (0~1000 μM) for 1, 2, 4 and 6 h, and the cell viability was analyzed by MTT assay. (**B**) Cells were treated with 400 μM H_2_O_2_ for the indicated times (0, 1, 4, 6, and 12 h), and the cells were stained with DCFH-DA to detect the intracellular ROS production in different times by flow cytometry. (**C**) Cells were treated with 0, 400 and 800 μM H_2_O_2_ for 4 h, and apoptosis was determined by flow cytometry followed by Annexin V–PI double staining. (**D**) Cells were treated with 400 μM H_2_O_2_ for 4 h, and the cell morphology was observed using an inverted/phase-contrast microscope. Data represent means ± S.E.M of three independent experiments and differences between mean values were assessed by one-way ANOVA. ^*^p < 0.01 indicates the significant difference compared with control group.

The results showed that the viability of MC3T3-E1 cells exposed to H_2_O_2_ was decreased in both time- and dose- dependent manners (Figure 1A). MC3T3-E1 cells were stained with DCFH-DA to assess the effect of H_2_O_2_ on the intracellular ROS production. Intracellular ROS production significantly increased with passage of time in H_2_O_2_-treated MC3T3-E1 cells (Figure 1B). Flow cytometric analysis demonstrated that the apoptotic osteoblasts increased with the increase of the dose of H_2_O_2_ (Figure 1C). As observed under microscope, treatment with H_2_O_2_ (400 μM) for 4 h resulted in significant cell shrinkage and a decrease in the rate of cellular attachment compared to the control group (Figure 1D). According to the results, the concentration (400 μM) of H_2_O_2_ for 4 h was chosen to be the model condition of oxidative stress in osteoblast cells for further research.

### CGA promotes MC3T3-E1 cells proliferation

**Figure 2:**
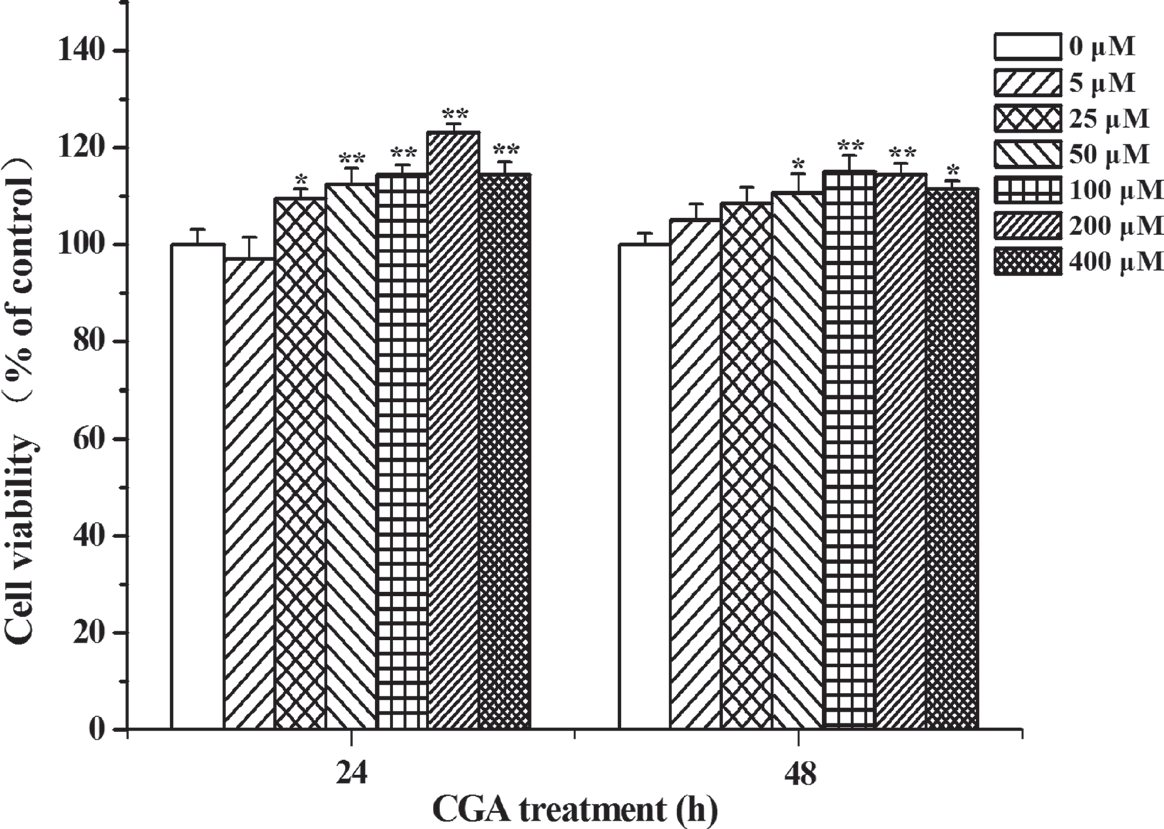
Effects of CGA on cell viability Effect of CGA on the viability of MC3T3-E1 cells was measured using MTT assay. Cells were treated with various concentrations (0, 5, 25, 50, 100, 200, 400 μM) of CGA for 24, 48 h. Data represent means ± S.E.M of six separate experiments and differences between mean values were assessed by one-way ANOVA. *p < 0.05 and ^*^p < 0.01 indicate the significant difference compared with control group.

Cell viability was tested after being treated with different concentrations (0, 5, 25, 50, 100, 200, 400 μM) of CGA for 24 and 48 h. Various concentrations of CGA had a marked role in promoting cell proliferation without cytotoxicity, and this effect was in a time- and dose-dependent manner in the range of 25 to 400 μM (Figure 2).

### CGA improves cell viability and reduces MC3T3-E1 apoptosis after H_2_O_2_ exposure

**Figure 3:**
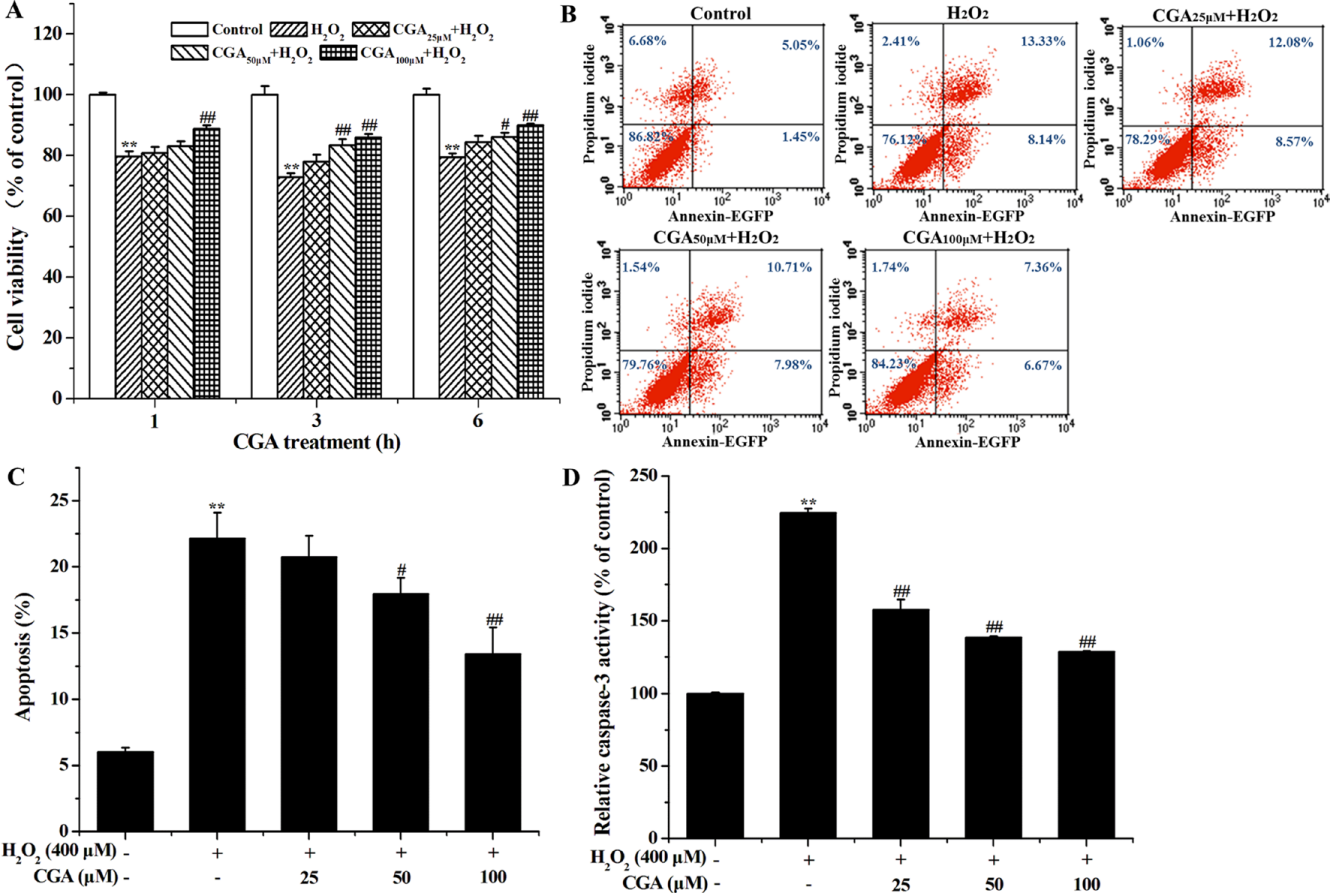
Protective effect of CGA on H_2_O_2_-induced cytotoxicity and inhibitory effect of CGA on H_2_O_2_-induced apoptosis in MC3T3-E1 cells (**A**) Cells were pretreated with or without CGA at the indicated concentrations for 1, 3, 6 h and then incubated in the presence of H_2_O_2_ (400 μM). The cell viability was determined by MTT assay. (**B**) Cells were pretreated with or without CGA at the indicated concentrations for 3 h before treatment with H_2_O_2_ (400 μM). Apoptosis was measured by flow cytometry, followed by Annexin V-EGFP (FL 1 channels) and PI (FL 2 channels) double staining. (**C**) The percentage of apoptosis was counted including early apoptosis (in the lower-right quadrants) and late apoptosis (in the upper-right quadrant). (**D**) The activity of caspase-3 in cell lysates was measured using respective substrate peptide Ac-DEVD-ρNA. Data represent means ± S.E.M of three independent experiments and differences between mean values were assessed by one-way ANOVA. *p < 0.05 and ^*^p < 0.01 indicate the significant difference compared with control group; ^#^*p* < 0.05 and ^##^*p* < 0.01 indicate the significant difference compared with H_2_O_2_-treated group.

The results demonstrated that H_2_O_2_ exposure markedly reduced cell viability, which was attenuated by CGA treatment (Figure 3A). The results of cell apoptosis detection by flow cytometry using Annexin V/PI double staining showed that after exposure to H_2_O_2_ for 4 h, CGA lowered apoptosis in a dose-dependent manner (Figure 3B, 3C). Compared with the control group, exposure to 400 μM H_2_O_2_ for 4 h activated caspase-3 of the MC3T3-E1 cells. CGA treatment was found to reduce caspase-3 activity in a dose-dependent manner (Figure 3D). This result suggests that CGA inhibited caspase 3-mediated cell apoptosis induced by H_2_O_2_.

### CGA inhibits H_2_O_2_-induced oxidative stress in MC3T3-E1 cells

**Figure 4:**
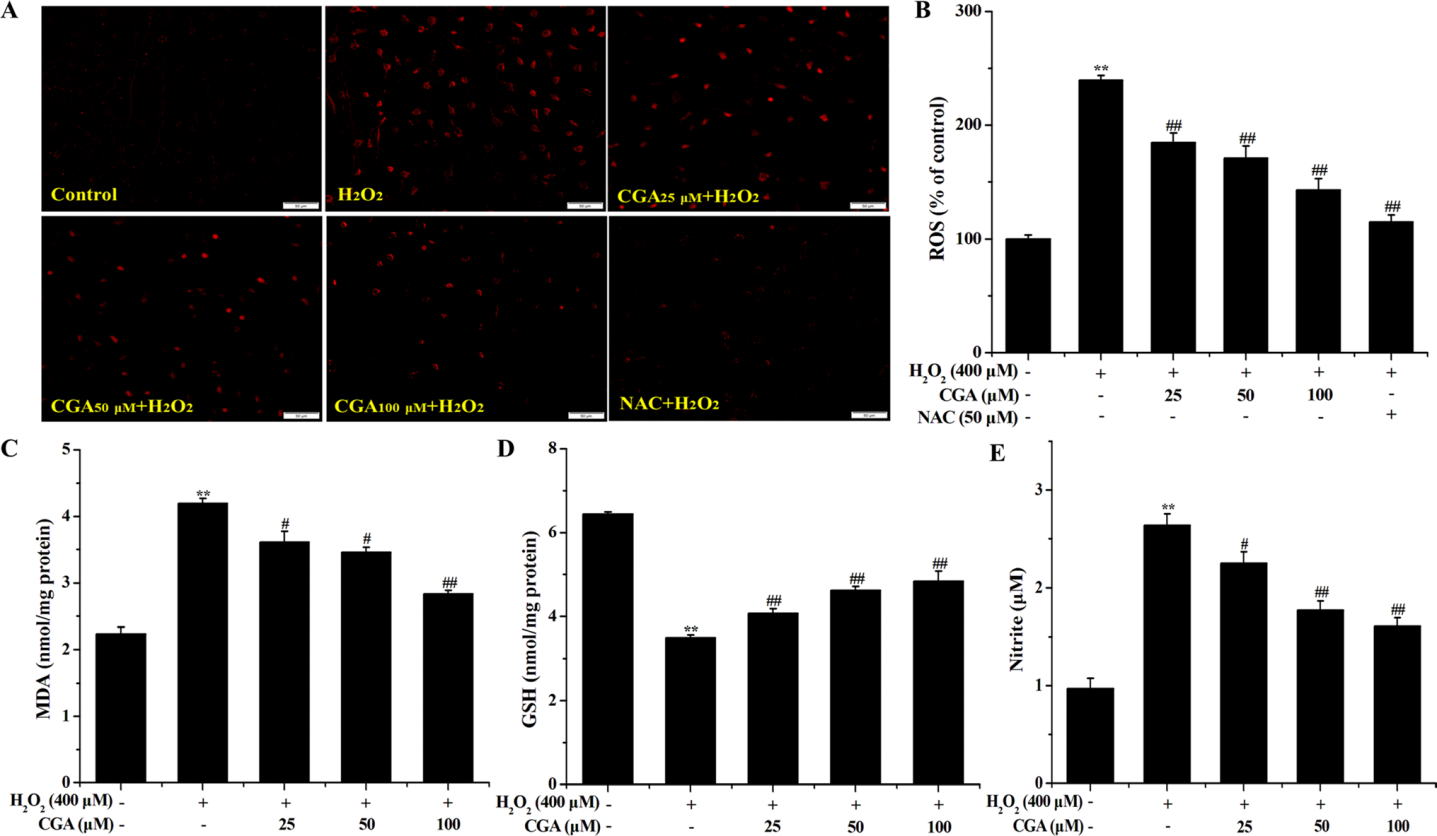
Effects of CGA on intracellular ROS, MDA, GSH and NO levels after H_2_O_2_ treatment (**A**) and (**B**) MC3T3-E1 cells were pretreated with or without CGA at the indicated concentrations in the presence or absence of 50 μM NAC for 1 h before treatment with 400 μM H_2_O_2_, ROS generation was observed using fluorescence microscopy and the fluorescence intensity of ROS was measured by a fluorescence microplate reader. (**C**), (**D**) and (**E**) Cells were pretreated with or without CGA at the indicated concentrations for 3 h and incubated in the presence of H_2_O_2_ (400 μM). Data represent means ± S.E.M of three independent experiments and differences between mean values were assessed by one-way ANOVA. *p < 0.05 and ^*^p < 0.01 indicate the significant difference compared with control group; ^#^*p* < 0.05 and ^##^*p* < 0.01 indicate the significant difference compared with H_2_O_2_-treated group.

Treatment of MC3T3-E1 cells with H_2_O_2_ alone clearly increased the levels of ROS, MDA and NO, and decreased GSH content. In contrast, pre-treatment with CGA significantly reversed the effect of H_2_O_2_-induced ROS, MDA, GSH (Figure 4A–4D), and NO level (Figure 4E) in a dose-dependent manner, respectively.

### CGA increases HO-1 expression and it protects oxidative stress in MC3T3-E1 cells

**Figure 5:**
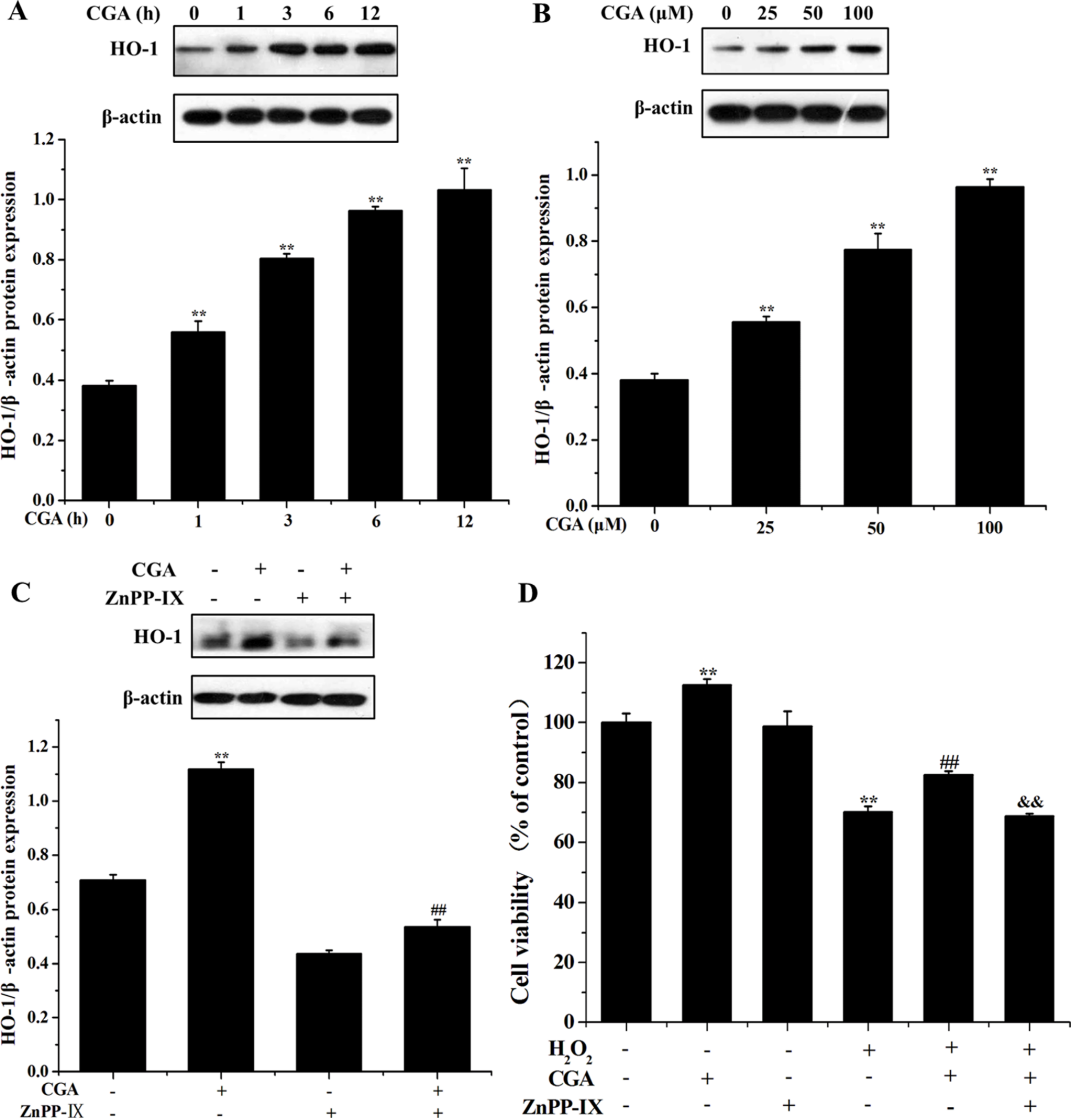
Effects of CGA on HO-1 protein induction (**A**) Cells were treated with CGA (100 μM) for indicated time periods (0, 1, 3, 6 and 12 h). (**B**) Cells were treated with indicated concentrations (0, 25, 50 and 100 μM) of CGA for 3 h. Cell lysates were subjected to western blot analysis with anti-HO-1 antibody. (**C**) Cells were pretreated with ZnPP-IX (5 μM) for 1 h prior to incubation with CGA (100 μM) for 3 h and then proteins were extracted for western blot analysis using anti-HO-1 antibody. (**D**) Cells were pretreated with ZnPP-IX (5 μM) for 1 h, and then incubated with or without CGA (100 μM) for 3 h, followed by exposure of H_2_O_2_. Cell viability was measured using the MTT assay. ^*^p < 0.01 indicates the significant difference compared with control group; ^##^*p* < 0.01 indicates the significant difference compared with H_2_O_2_-treated group; ^&&^*p* < 0.01 indicates the significant difference compared with CGA-treated group.

Western blot analysis demonstrated that 100 μM CGA treatment significantly increased HO-1 protein level in a time-dependent manner (Figure 5A). Furthermore, cells were treated with various doses of CGA (0, 25, 50, 100 μM) for 3 h, and CGA concentration -dependently, increased HO-1 protein expression (Figure 5B). ZnPP IX, a HO-1 inhibitor, significantly reduced the expression of HO-1 induction by CGA (Figure 5C). The increased cell viability by CGA after exposure to H_2_O_2_ was significantly inhibited by ZnPP IX (Figure 5D), indicating that HO-1 induction is responsible for increased cell viability with CGA-pretreated cells under oxidative stress condition.

### CGA increases Nrf2 nuclear translocation and activates PI3K/Akt pathway in MC3T3-E1 cells

**Figure 6:**
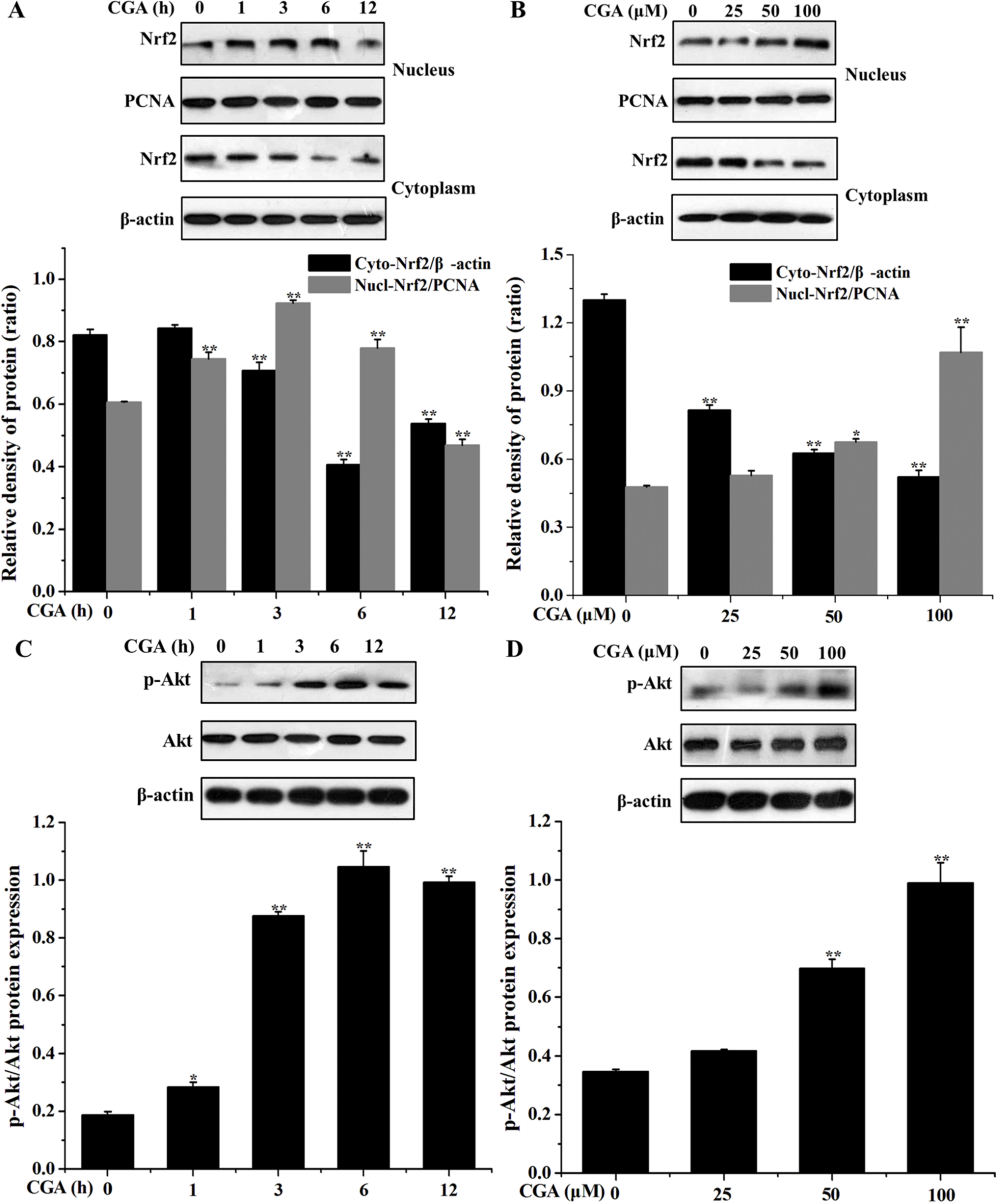
Effects of CGA on Nrf2 nuclear translocation and PI3K/Akt phosphorylation in MC3T3-E1 cells (**A**) and (**C**) Cells were treated with CGA (100 μM) for indicated time points (0, 1, 3, 6 and 12 h). (**B)** and (**D**) Cells were treated with indicated concentrations (0, 25, 50 and 100 μM) of CGA for 3 h. (A) and (B) The nuclear and cytosolic levels of Nrf2 were determined by western blot analysis. PCNA was used as nuclear loading control. (C) and (D) Cell lysates were subjected to western blot analysis with anti-Akt or p-Akt antibodies. β-actin was used as loading control. ^*^p < 0.01 indicates the significant difference compared with control group.

Western blot analysis showed that nuclear Nrf2 protein expression increased at 1 h, 3 h, and continued to rise up to 6 h, whereas Nrf2 protein in the cytoplasm decreased at 3 h, 6 h, and continued to rise up to 12 h compared with the control group (Figure 6A). CGA concentration-dependently induced translocation of Nrf2 from cytosol to nucleus (Figure 6B). Our data showed that CGA treatment notably enhanced Akt phosphorylation in a dose- and time-dependent manner, while no significant changes were found in the total Akt protein level (Figure 6C, 6D). These finding suggested that CGA-induced HO-1 expression and Nrf2 nuclear translocation, which may be associated with PI3K/Akt signaling pathway

### Role of PI3K/Akt on CGA-induced HO-1 expression and Nrf2 nuclear translocation in MC3T3-E1 cells

**Figure 7:**
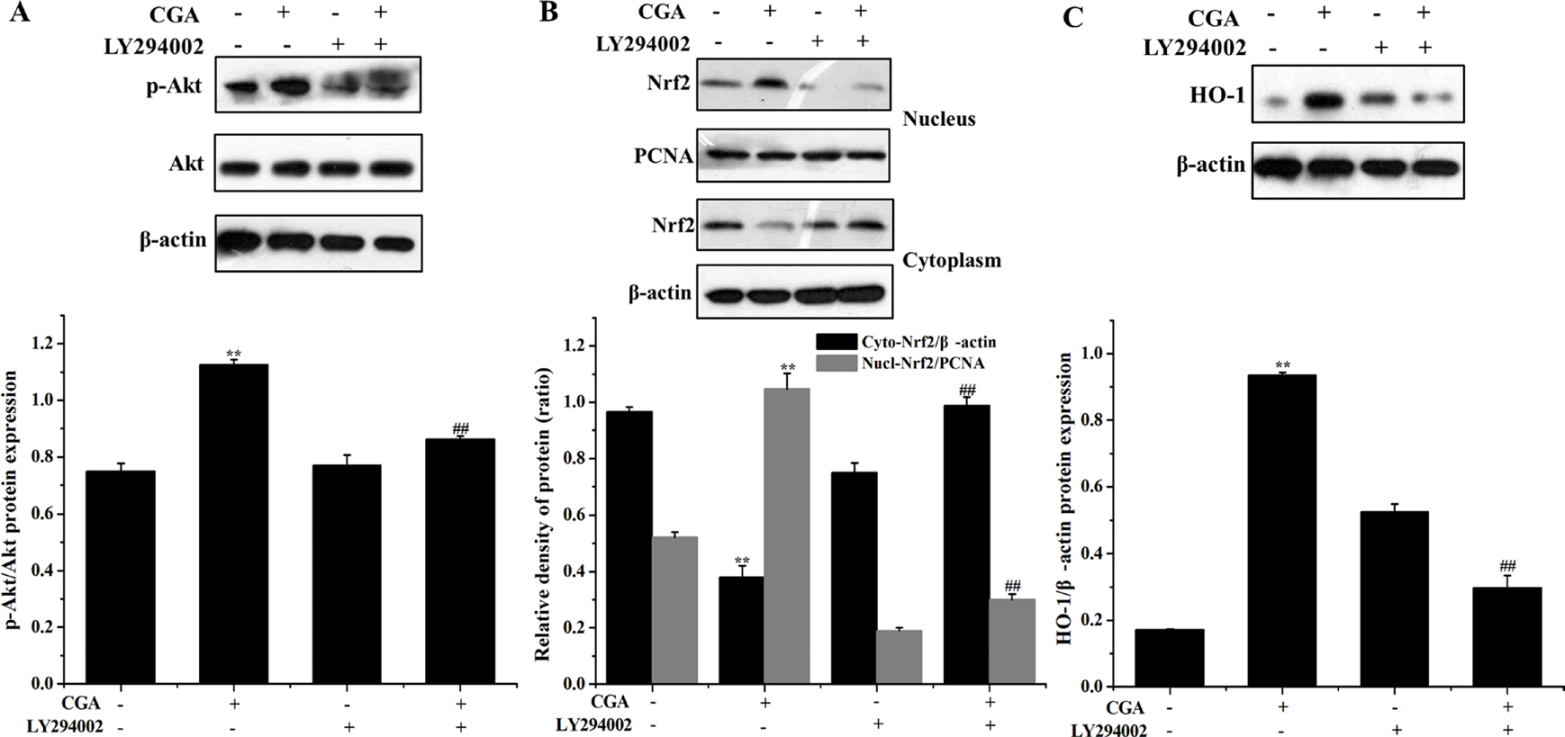
Role of the PI3K/Akt pathway in CGA-induced Nrf2 transcription and HO-1 expression (**A**), (**B)** and (**C**) Cells were pretreated with 20 μM LY294002 for 1 h, and then treated with 100 μM CGA for 3 h. Nrf2, p-Akt and HO-1 levels were determined by western blot analysis. ^*^p < 0.01 indicates the significant difference compared with control group; ^##^*p* < 0.01 indicates the significant difference compared with H_2_O_2_-treated group.

Our results revealed that inhibition of the PI3K/Akt pathway significantly suppressed Akt phosphorylation and the nuclear translocation of Nrf2 induced by CGA (Figure 7A, 7B). Similarly, pharmacological inhibition of PI3K/Akt significantly suppressed the protein level of HO-1 induced by CGA (Figure 7C).

### Role of PI3K/Akt pathway on cytoprotection induced by CGA in MC3T3-E1 cells

**Figure 8:**
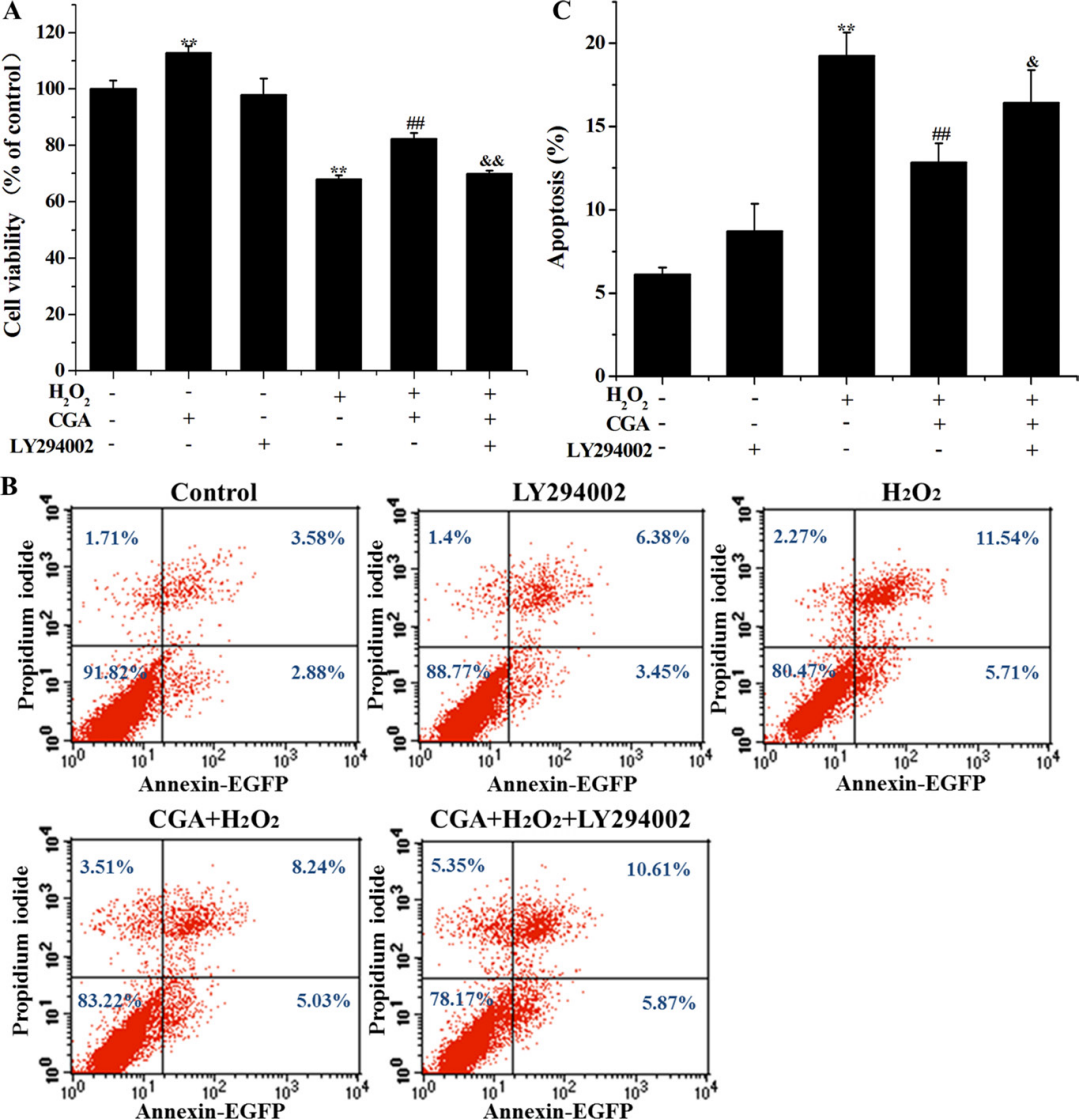
Effects of CGA and selective inhibitor LY294002 on H_2_O_2_-induced cytotoxicity and apoptosis in MC3T3-E1 cells Cells were pretreated with 20 μM LY294002 for 1 h, and then incubated with or without CGA (100 μM) for 3 h, followed by exposure with H_2_O_2_. (**A**) Cell viability was measured using the MTT assay. (**B)** and (**C**) Cell apoptosis was determined by the Annexin V-EGFP/PI staining assay. ^*^p < 0.01 indicates the significant difference compared with control group; ^##^*p* < 0.01 indicates the significant difference compared with H_2_O_2_-treated group; ^&^*p* < 0.05 and ^&&^*p* < 0.01 indicate the significant difference compared with CGA-treated group.

LY294002, an inhibitor of PI3K/Akt, abolished the protective effect of CGA against H_2_O_2_-induced decreased cell viability and increased apoptotic rate (Figure 8), indicating that the protective role of CGA was through the PI3K/Akt pathway, a vital upstream signaling pathway that mediated the Nrf2-related cytoprotective effect.

## DISCUSSION

Several observational studies showed that ROS production reduces the bone formation of osteoblastic cells, decreases bone mass and alters bone micro-architecture in age-related osteoporosis or in secondary osteoporosis [1, 4, 15]. H_2_O_2_, as involvement of oxidative stress, may induce the self-generation of free radicals and produce a wide range of damages including apoptosis, necrosis or autophagy of various types of cells, including osteoblast cells [26–28]. In this study, MC3T3-E1 cells were exposed to H_2_O_2_ to perform a model of oxidative damage, we found that treating MC3T3-E1 cells with H_2_O_2_ resulted in reduction of cell viability, ROS production and apoptosis. Moreover, bone pathophysiology is highly sensitive to ROS production, contributing to define the cellular and molecular basis of the biological activities of ROS [29]. Therefore, it is tempting to suggest that exogenous administration of antioxidants should be investigated as a potential approach to protect cells against ROS for the treatment of osteoporosis. Previous studies have revealed the antioxidant effect of CGA, effectively scavenging free radicals and inhibiting lipid peroxidation [25, 30, 31]. Recent report demonstrated that CGA efficiently inhibits osteoblast apoptosis and ameliorates hormone-induced femoral head necrosis [32]. Therefore, the protective effects and underlying mechanisms of CGA against oxidative damage were explored in the current study. The MTT colorimetric assay showed that CGA can increase the proliferation capabilities of osteoblasts. Moreover, the results showed that CGA significantly attenuated H_2_O_2_-induced cell damage through increasing cell viability, decreasing apoptotic rate and caspase-3 activity in MC3T3-E1 cells.

MDA is frequently used to measure the degree of lipid peroxidation, which is closely related to cell damage [33]. GSH is a vital antioxidant that scavenges hydrogen peroxide and ROS [34]. When excessively produced, NO may react with superoxide anion radicals, leading to the strong oxidant, peroxynitrite, and destroying functional tissues [35]. In the current study, CGA significantly decreased ROS and NO production, and the content of MDA, while increasing the level of GSH in MC3T3-E1 cells. These findings indicated that blocking H_2_O_2_-induced apoptosis by CGA may be mediated by an increase in antioxidant capacity, and/or the prevention of oxidative stress.

HO-1, one of the pivotal cytoprotective enzymes in cellular defense, is a key protein that plays an important role in the cellular adaptation to oxidative stress inflicted by pathological events in a wide variety of cells [7, 8, 12, 36]. Many investigations have confirmed that upregulation of HO-1 contributes to the cellular defense mechanism in response to oxidative insults [20, 34, 37]. The present study showed that CGA treatment significantly upregulated HO-1 protein expression in a dose- and time-dependent manner, indicating that CGA exhibited antioxidant activity by upregulation HO-1 expression. Moreover, inhibition of HO-1 can partially reverse the increased cell viability induced by CGA in the presence of H_2_O_2_. These data clearly demonstrated that the increased HO-1 protein expression in MC3T3-E1 cells is responsible for CGA-induced protective effect against H_2_O_2_-induced cytotoxicity, implicating that CGA confers protection against oxidative stress by increasing HO-1 levels.

Nrf2 is known as an upstream mediator of ARE-dependent phase II enzyme expression [38]. The Nrf2 translocation has been further demonstrated to up-regulate the expression of antioxidant genes [7, 36, 37], which plays an essential role in the induction of HO-1 and may provide a useful therapeutic approach to combating oxidative stress. Furthermore, current study reveals that the induction of HO-1 expression is mediated by Nrf2 activation in MC3T3-E1 cells [18]. Western blot analysis depicted that CGA treatment significantly increased Nrf2 protein level in the nucleus from 1 h to 6 h and in a dose range of 50 μM to 100 μM, which suggested that CGA may up-regulate HO-1 protein expression by activating Nrf2 signaling pathway. When MC3T3-E1 cells were treated with CGA for a long period time (12 h), nuclear Nrf2 dramatically decreased, which indicated that Nrf2 anti-oxidative stress pathway just adaptively responded to CGA treatment in a short time.

Furthermore, PI3K/Akt signaling pathway is commonly involved in HO-1 expression and in Nrf2-dependent transcription in diverse cell types in response to oxidative stress and different stimuli [7, 33, 37, 39]. Therefore, the current experiments were designed to determine a role for PI3K/Akt as a possible regulator of cell survival in CGA-induced HO-1 expression. In the present findings, CGA was found to increase the phosphorylation level of Akt protein, suggesting that enhancement of Akt protein phosphorylation may contribute to the CGA-induced HO-1 expression. However, LY294002, a specific PI3K/Akt inhibitor, significantly suppressed the nuclear localization of Nrf2 and markedly blocked HO-1 protein expression in the presence of CGA. Moreover, inhibition of PI3K/Akt signaling pathway partially reverse the increased cell viability and decreased apoptotic rate induced by CGA in the presence of H_2_O_2_, it indicating that PI3K/Akt pathway plays a central role in the mechanism of CGA-induced Nrf2/HO-1 activation.

**Figure 9:**
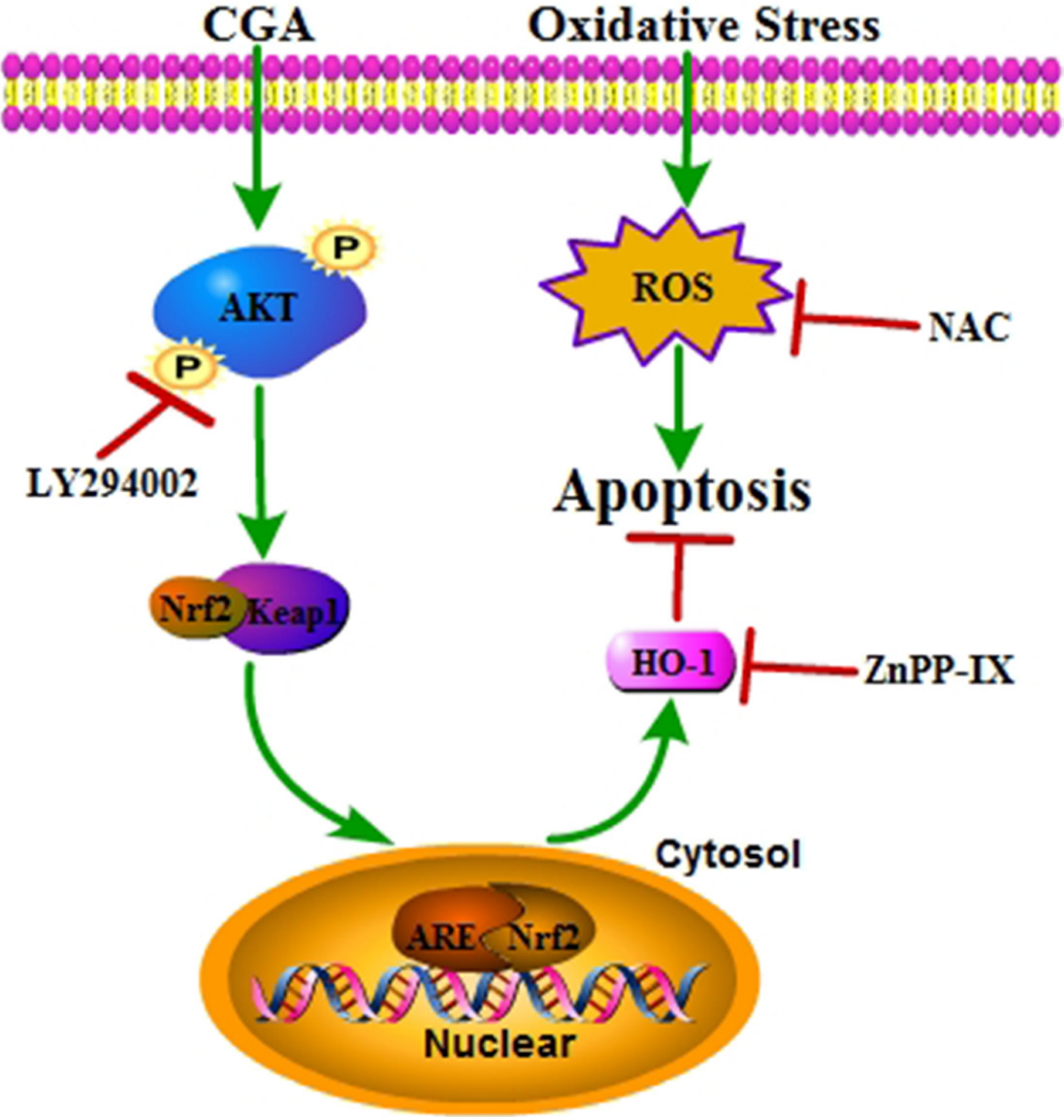
A proposed signaling pathway involved in CGA against H2O2-induced oxidative damage Schematic diagram shows that CGA induces Nrf2 –mediated cytoprotective protein via activation of PI3K/Akt signaling, which protects against oxidative stress of osteoblast cells. H2O2 induced ROS generation resulting in cell apoptosis. Meanwhile, CGA activates Nrf2/HO-1 through the PI3K/Akt pathway. Activation of Nrf2/HO-1 protects against H2O2 –induced osteoblast apoptosis. Green arrow indicates stimulation and red bar indicates inhibition.

Our findings demonstrated that CGA activates nuclear translocation of Nrf2, induces the expression of HO-1, and reduces H2O2-induced cell death *in vitro* via activating PI3K/Akt pathway in MC3T3-E1 cells as shown in Figure 9. However, it has to be emphasized that whether there is the same effect of CGA *in vivo* or in other cell lines. Moreover, further investigations will be carried out in the future to reveal that whether other redox-sensitive transcription factors and targets, such as activator protein-1 (AP-1) and nuclear factor-κB (NF-κB) [8, 16], and other possible pathways, such as mitogen-activated protein kinase (MAPK) and AMP- activated protein kinase (AMPK) [8, 34, 40, 41], may participate in the process of CGA-mediated antioxidant effect.

## MATERIALS AND METHODS

### Reagents and antibodies

CGA [> 96% high-performance liquid chromatography (HPLC) purity] was purchased from National Institutes for Food and Drug Control (Beijing, China). 3- (4, 5-dimethyl-thiazol-2-yl)-2,5-dipheny l tetrazolium bromide (MTT), dimethyl sulfoxide (DMSO) and Zinc protoporphyrin IX (ZnPP) were purchased from Sigma-Aldrich Chemical (Sigma, USA). Minimum essential medium (α-MEM) and fetal bovine serum (FBS) were purchased from Gibco (Grand island, NY, USA). Primary antibodies to HO-1 (Cat # ab13248), Nrf2 (Cat # ab62352) and corresponding secondary antibodies (Cat # ab6721, Cat # ab6728) were obtained from Abcam (Abcam, USA). Antibodies for β-actin (Cat # AC004) and proliferating cell nuclear antigen (PCNA, Cat # A0264) were purchased from ABclonal Technology (Wuhan, China). LY294002 (Cat # 9901) and antibodies against Akt (Cat # 4685), and p-Akt (Cat # 4060) were purchased from Cell Signaling Technology (Danvers, MA, USA).

### Cell culture and treatment

The mouse osteoblastic cell line MC3T3-E1 was purchased from American Type Culture Collection (ATCC, Rockville, MD, USA). Cells were cultured in α-MEM medium supplemented with 10% FBS and 1% antibiotics (100 U/mL penicillin and 100 U/mL streptomycin) at 37°C in a humidified atmosphere of 5% CO_2_. All cells were washed with phosphate buffer saline (PBS, PH 7.2–7.4) before incubation with CGA or H_2_O_2_. After reaching 85% confluence, the cells were treated with medium containing different concentrations of CGA (0, 25, 50, 100 μM) for 1, 3, 6 h before treatment with H_2_O_2_ (400 μM) for 4 h. ZnPP IX (5 μM) or LY294002 (20 μM) was treated for 1 h before CGA treatment. CGA, ZnPP IX or LY294002 were dissolved in DMSO, and the final DMSO concentration was ≤ 0.1% (v/v).

### Cell viability assay

Cell viability was determined by MTT reduction assay. In brief, MC3T3-E1 cells were pre-incubated with α-MEM containing 10% FBS overnight in 96-well plates at a density of 5 × 10^4^ cells per well. According to the experimental design, treated cells in each well were added with α-MEM solution containing 10% MTT. The cells were incubated at 37°C for 4 h, the supernatants were removed, and the formazan crystals were dissolved in 150 μl DMSO. Absorbance was recorded at a wavelength of 490 nm and reference wavelength of 630 nm using a microplate reader (Bio-Rad, Foster, California, USA).

### Assay of Annexin V-EGFP/PI apoptosis

Cellular apoptosis was determined using the Annexin V-EGFP/PI Cell Apoptosis Detection Kit (KeyGEN, Nanjing, China). Briefly, cells were plated into 6-well plates and treated with LY294002, CGA or H_2_O_2_ according to the experimental design. Following each specific treatment, cells were harvested, then washed twice with ice-cold PBS and centrifuged at 1000 rpm for 10 min. Cells were resuspended in 100 μl of 1× binding buffer and transferred to sterile flow cytometry glass tubes 5 μl of Annexin V-EGFP and 5 μl of propidium iodide were added and incubated at room temperature (25°C) in dark conditions for 15 min. Finally, detection was performed using flow cytometric analysis according to manufacturer's instructions (Beckman, Fullerton, California, USA).

### Measurement of ROS production

The level of ROS was detected by measuring intracellular ROS formation using a ROS detection kit (Beyotime, Haimen, China). Briefly, MC3T3-E1 cells, cultured in 6-well plates for 24 h before treatment, were pretreated with CGA (0, 25, 50, 100 μM) and then exposed with H_2_O_2_ (400 μM) for 1 h to induce ROS production. Cells were washed twice with PBS and then incubated with 5μM 2′, 7′- dichlorofluorescein diacetate (DCFH-DA) for 30 min. Fluorescence staining was visualized using a fluorescence microscope (Olympus, IX71), and fluorescence assays were measured and quantified using a fluorescence microplate reader (Tecan, Sunrise) at excitation/emission 525/610 nm and a flow cytometer (Beckman, Fullerton, California, USA) at excitation/emission 488/525 nm.

### Determination of caspase-3 activity

MC3T3-E1 cells were cultured in 6-well plates and then treated with CGA (0, 25, 50, 100 μM) for 3 h prior to 400 μM H_2_O_2_ treatment for 4 h. In brief, treated cells were extracted using lysis buffer (Beyotime, Haimen, China), and then the supernatants were collected and incubated with the substrate Ac-DEVD-*p*NA (acetyl-Asp-Glu-Val-Asp *p*-nitroanilide), which produces yellow *p*NA. Absorbance was measured in a microplate reader (Bio-Rad, Foster, California, USA) at a wavelength of 405 nm.

### Analysis of MDA, GSH and NO

MC3T3-E1 cells were treated with different concentration of CGA (0, 25, 50, 100 μM) for 3 h, after which H_2_O_2_ (400 μM) was added for 4 h. The cells in each group were lysed and prepared to determine MDA and GSH contents using commercial colorimetric assay kits (Beyotime, Haimen, China) according to manufacturer's instructions. In addition, culture supernatants were collected and assayed to measure NO release using a NO assay kit (Beyotime, Haimen, China). The results were calculated with reference to a standard curve and expressed as nM/mg protein for MDA and GSH and μM for NO, respectively.

### Western blot analysis

MC3T3-E1 cells were treated with the indicated agents according to the experimental design. Total cellular protein of MC3T3-E1 cells was extracted using a total protein extraction kit (KeyGEN, Nanjing, China), and nuclear and cytoplasmic extractions were prepared using a nuclear and cytoplasmic extraction kit (KeyGEN, Nanjing, China), respectively. The protein content was quantified using a BCA protein assay kit (Beyotime, Haimen, China) according to the manufacturer's instructions. Proteins were separated by sodium dodecyl sulphate polyacrylamide gel electrophoresis (SDS-PAGE) and transferred to a poly-vinylidine difluoride membrane (PVDF, Millipore, MA, USA). After being blocked, the membranes were incubated over night at 4°C with specific primary antibodies. Then membranes were probed with HRP-conjugated secondary antibody for 1 h at room temperature and detection was performed by an enhanced chemiluminescence system (ECL, Beyotime, Haimen, China). The results were normalized to β-actin or PCNA and analyzed using Image J (National Institutes of Mental Health, Bethesda, MD, USA).

### Statistical analysis

Results from a representative of three independent experiments were expressed as means ± standard error of the mean (SEM), and assessed by the one-way analysis of variance (ANOVA) followed by least significant difference (LSD) test for multiple comparisons, and post hoc test using SPSS 19.0 (SPSS, Inc., Chicago, IL, USA). A value of *p* < 0.05 was considered statistically significant.

## CONCLUSIONS

In summary, our results showed that CGA protected MC3T3-E1 cells against H_2_O_2_-induced caspase 3-dependent cell death by decreasing ROS, MDA and NO production, while increasing GSH content, it also demonstrated that CGA augments the cellular antioxidant defense capacity by enhancing Nrf2 nuclear translocation and upregulating HO-1 induction, and that the PI3K/Akt signaling pathway plays a central role in this mechanism, thereby protecting osteoblasts from oxidative damage.
